# Biomarkers Potency to Monitor Non-target Fauna Poisoning by Anticoagulant Rodenticides

**DOI:** 10.3389/fvets.2020.616276

**Published:** 2020-12-23

**Authors:** Antoine Rached, Meg-Anne Moriceau, Xavier Serfaty, Sebastien Lefebvre, Virginie Lattard

**Affiliations:** USC 1233 RS2GP, VetAgro Sup, INRA, University of Lyon, Marcy l'Etoile, France

**Keywords:** biomarkers, rodenticides, primary exposure, secondary exposure, non-target, antivitamin K anticoagulant, toxicokinetics

## Abstract

The widespread use of pesticides to control agricultural pests is a hot topic on the public scene of environmental health. Selective pest control for minimum environmental impact is a major goal of the environmental toxicology field, notably to avoid unintended poisoning in different organisms. Anticoagulant rodenticides cause abnormal blood coagulation process; they have been widely used to control rodents, allowing inadvertent primary and secondary exposure in domestic animals and non-target predatory wildlife species through direct ingestion of rodenticide-containing bait or by consumption of poisoned prey. To report toxic effect, the most common approach is the measurement of liver or plasma residues of anticoagulant rodenticides in dead or intoxicated animals showing clinical symptoms. However, one major challenge is that literature currently lacks a hepatic or plasma concentration threshold value for the differentiation of exposure from toxicity. Regarding the variation in pharmacology properties of anticoagulant rodenticides inter- and intra-species, the dose-response relationship must be defined for each species to prejudge the relative risk of poisoning. Beyond that, biomarkers are a key solution widely used for ecological risk assessment of contaminants. Since anticoagulant rodenticides (AR) have toxic effects at the biochemical level, biomarkers can serve as indicators of toxic exposure. In this sense, toxicological knowledge of anticoagulant rodenticides within organisms is an important tool for defining sensitive, specific, and suitable biomarkers. In this review, we provide an overview of the toxicodynamic and toxicokinetic parameters of anticoagulant rodenticides in different animal species. We examine different types of biomarkers used to characterize and differentiate the exposure and toxic effects of anticoagulant rodenticide, showing the strengths and weaknesses of the assays. Finally, we describe possible new biomarkers and highlight their capabilities.

## Introduction

Pesticides are today the main means of pest control, making it possible to mitigate the economic, environmental and health consequences when pest population outbreak occurs (1). Nevertheless, legitimate environmental concerns relative to the use of all kind of pesticides (insecticides, herbicides, fungicides, rodenticides, fumigants) are growing. One of this concern involves their impact on non-target living species since more than 50 years already. A central question on this topic is: “How to differentiate sub-toxic exposure to sublethal effects or lethal exposure?” (2–5). Indeed, in order to monitor the ecotoxicity of molecules and their effects on non-target wildlife and thus adapt pesticide use practices, it is necessary to clearly establish the causal link between the death of a non-target species or negative fitness effects on non-target species and pesticide exposure. Due to the diversity of non-target species, from small mammals to birds of prey, with for each species particular sensitivities or resistances to the pesticide in question, it is difficult to establish a level of exposure that may lead to the death. Nevertheless, for animal species, one powerful way to answer this question is through detection and/or quantification of biomolecules or compounds characteristic of a non-toxic or a toxic exposure, the so-called biomarkers, associated with clinical signs and near-environmental analysis. In this review, we will focus on summarizing current knowledge on biomarkers and associated parameters differentiation of sub-lethal exposure effects vs. lethal exposure for anticoagulant rodenticides (AR), that are also a hot topic of environmental concerns since decades (6–9).

More and more primary or secondary non-target intoxications with anticoagulant rodenticides are reported in the literature including mostly birds, especially raptors, but also a minor part of domestic animals such as cats, dogs and horses, and other wildlife species such as dears, polecats, martens, foxes, and very relevant literature reviews are already available on this topic (10–13). Few studies deal with determination of parameters of poisoning in different species (14) and the way to treat non-target poisoned animals is still relatively obscure while many questions on this topic still need answers, such as how long does the treatment should last, what is the frequency and the quantity of vitamin K needed as a function of the species or even based on the race (15). From this lack arise many questions about the follow up of AR and their impact on environment, notably: how to attribute the cause of death of an AR exposed animal? Considering the diversity of exposed species with specific susceptibility to AR and the diversity of AR molecules with different pharmacodynamic and pharmacokinetic parameters and the frequent detection of multiple contaminants for the same animal, the simple level of exposure of the animals seems to be insufficient to point at AR as sole responsible of the death. No literature review on the specific biomarkers of the sub-lethal or lethal exposure to anticoagulant rodenticides in non-target species is available until now. The aim of this review is to outline existing and propose new biomarkers, in order to allow a better follow-up of anticoagulant exposure and intoxication.

## Anticoagulant Rodenticides use and Properties

### Importance of Anticoagulant Rodenticides Use and Animal Exposure

#### Use of Anticoagulant Rodenticides

Rodents control using anticoagulant rodenticides was introduced officially in 1948 (16), answering to the demand of authority, general population (17, 18) and mostly farmers and of all the production and supply chains against tremendous damage caused by rodents all around the world, sometimes resulting in the loss of 100% of a production (19, 20). Anticoagulant rodenticides provide strong advantages among all chemical methods, e.g., they are slow acting compounds, they have a safe and very common antidote, the vitamin K, and they can be used at low concentration.

The risk of a pesticide used to control target species by causing their death is to select living target species that are less or not sensitive to this pesticide, the latter specimens are said “resistant.” In 60's; the first resistant rodent population has been described (21). To deal with the emergence of resistant rodent populations resulting from the use of these first generation of anticoagulant rodenticides (FGAR) (warfarin, diphacinone, chlorophacinone) a second generation of anticoagulant rodenticides (SGAR) has been developed (22–24). Second generation molecules (bromadiolone, brodifacoum, dicoumarol, difenacoum, difethialone…) are more efficient against resistant rodents and effective at a lower dose. Despite these abilities, resistance to some SGARs has been reported in different areas due to massive use (25, 26). Currently this second generation is the most used in developed countries.

Anticoagulant rodenticides are included in baits of different types and forms (grains, pellets, solid blocks) depending on the application site and the targeted rodents (27). The presence of a carbohydrate source in bait formulation is important to ensure high palatability to attract rodents. Unfortunately, they are also palatable to other non-target species, even if a bittering agent is usually added to the bait. Besides the physical and chemical properties of bait formulations, its consumption depends on the affluence of traditional food sources that interest rodents (28–30). In European Union, rodenticide baits must be disposed in secured bait station since 2013. These bait stations are supposed to avoid primary exposure of non-target species by physically preventing the access to baits and their spread through water (27, 31, 32). However, their use by amateurs is not mandatory and non-target animals having similar size to target rodents can still access to the bait.

#### Animal Exposure to Anticoagulant Rodenticides

##### Primary and secondary exposure to anticoagulant rodenticides

There are two types of exposures to consider. Primary exposure occurs when a non-target animal directly eats the bait, while secondary exposure occurs when a predator ingests preys previously exposed to AR. Prevention is different for each case. Considering the primary exposure, the goal is to avoid the access to the bait by ensuring their correct storage and using them in secured bait station as previously discussed. Considering the prevention of the secondary exposure, the matter is more complex. Indeed, AR is a long-acting poison and targeted rodents may die within 2–6 days after ingestion. Moreover, rodenticides may influence gradually rodents' behavior. Actually, poisoned rodents become weakened with reduced appetite and motionless, lose their nocturnal disposition and positive thigmotactic behavior; they are therefore more prone to predation (33–35). Thus, to mitigate secondary exposure, AR have to be less persistent in the target animal body.

##### Domestic animal exposure to anticoagulant rodenticides

In domestic animals, the majority of exposures are primary ones. They are often the consequences of a misuse or an improper storage of baits allowing domestic animals to access to AR. According to a French veterinary poison control center (CNITV), AR exposures represent 10% of the total call to the center and dogs accounted for 82.8% of AR exposure incidents, followed by cats with <10% (36). The pet exposures are mainly accidental. Indeed, the few studies that assess the exposure of healthy dogs without AR intake mentioned by the owner show that they are not chronically exposed to AR. Indeed, <2% of dogs are positive to AR (37, 38). Cats would be more prone to secondary exposure to AR. However, to the knowledge of the authors only primary exposure cases have been observed and studied in cats (36, 39). For pets the incidence of exposure seems to be seasonal, with an increase at the beginning of autumn (36, 39). Considering livestock, the primary exposure is mainly due to an improper storage of baits that makes them reachable. The incidence of these exposures seems to be low compared to pets (<2.2% of AR exposure cases) (36). Nevertheless, livestock exposures raise public health concerns discussed below.

Since the majority of AR exposures are accidental, owners notice the event and treatment is administered before symptoms occur. According the CNITV data, SGAR are reported in more than 60% of exposure cases (in 22%, the AR molecules are not identified) (36). This incidence is due to the highest efficiency of SGAR compared to FGAR, that are now less used. Moreover, the number of cases started to decrease after 2013 with the regulatory obligation to use bait station.

Livestock can also be exposed and intoxicated to natural anti-vitamin K like dicoumarol after the ingestion of moldy clover fodder (40, 41) or like ferprenin and ferulenol after giant fennel intake (42, 43).

##### Wildlife exposure to anticoagulant rodenticides

Depending on their size and dietary regimen, wild animals can be primary or secondary exposed to AR. The mandatory use of secured bait stations for the application of AR during a biocidal use may decrease the primary exposure of mammals larger than rats although some large mammals like wild boars seem to be highly primary exposed to AR (60%) in suburban area (44). However, small rodents can still access to baits. Thus, in the 20 meters perimeter around bait boxes, about 50% of non-target small mammals can be exposed to AR (45). This exposure affects all *taxa* and decreases with the distance to the bait boxes (45, 46). The small mammal exposure is a concern for both small-mammal populations themselves (notably for endangered species) but also for their predators.

Predators can be secondary exposed to AR from target and non-target species, according to their diet (47). The exposure of birds of prey, foxes, racoon dog, marten, bobcat, or polecat varies from 20 to up to 90% (48–53). This prevalence is strongly linked to the proximity of an area of human activity such as urban areas or farms (51, 54, 55). Similar to domestic animals, wildlife predators are exposed mostly to SGAR. SGAR are composed of asymmetric molecules called stereoisomers, but predators like red foxes are more exposed to some stereoisomers than others (56). While predator exposure to AR is high, it is more rarely linked to their death (57–60). However, it raises many questions about the consequences of this chronic exposure on the wildlife. Some mitigation risk measures to protect predator fauna have been tried as the removal of dead rodents (61). However, new approaches are needed to mitigate efficiently the secondary exposure of predators. The design of new “eco-friendly” SGAR, based on the different properties of AR stereoisomers, might be interesting and is discussed below (62). Finally, some mammals and birds might also be exposed by eating invertebrates that would have entered the bait box (63).

### Anticoagulant Rodenticides Properties

#### General Mechanism of Action of Anticoagulant Rodenticides

**Figure 1:**
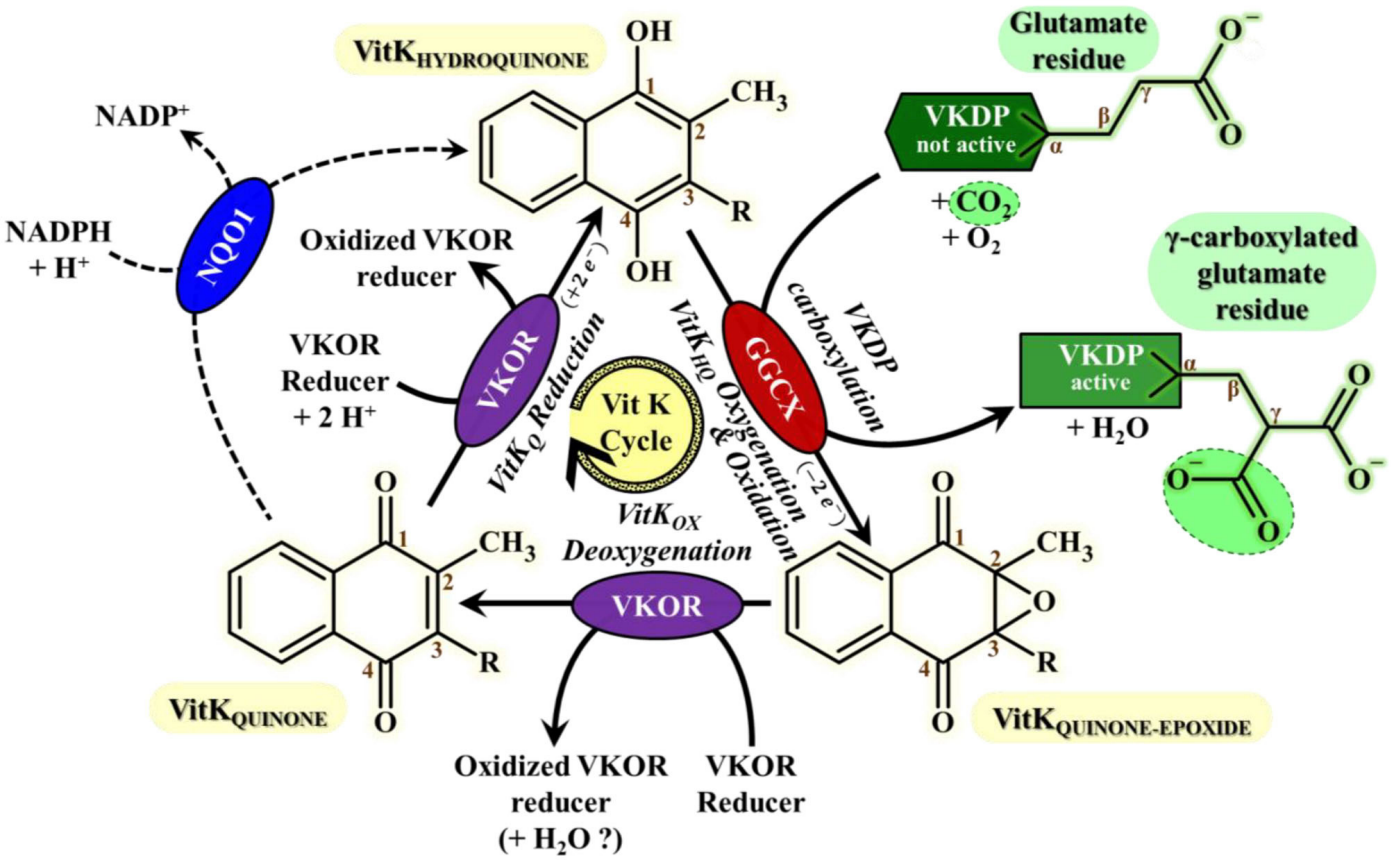
Vitamin K cycle most important features for VKAs mode of action understanding This cycle consists in the recycling of vitamin K HydroQuinone (VitK_HQ_) state, the sole active Vitamin K redox-state involved in the carboxylation mechanism of VKDPs catalyzed by the GGCX enzyme. The recycling of VitK_HQ_ needs the deoxygenation of Vitamin K Quinone-Epoxide state (VitK_OX_) only catalyzed by VKOR enzymes, followed by bielectronic reduction of Vitamin K Quinone state (VitK_Q_) mainly catalyzed by VKOR enzymes and by an alternative enzyme which current consensus is NQO1 (68). Gamma-Carboxylation of glutamates residues from Vitamin K Dependent proteins (VKDP) catalyzed by GGCX is required for activation of VKDPs proteins, including essential clotting factors. The regeneration of the reduction power of VKOR is sustained by a “VKOR reducer” that is much likely, as far as we know, a protein partner probably from PDI-like enzymes family. VKOR stands for VKORC1 or VKORC1L1; GGCX, Gamma-Glutamyl CarboXylase; NQO1, NADPH:Quinone Oxydoreductase 1; VKDP, Vitamin K-Dependent Protein; VitK_Q_, Vitamin K Quinone form; VitK_HQ_, Vitamin K Hyroquinone form; VitK_OX_, Vitamin K quinone-epoxide form.

Vitamin K in its hydroquinone form (VitK_HQ_) is a cofactor of Gamma-Glutamyl CarboXylase (GGCX) enzyme that performs a gamma-carboxylation of glutamate residue of some proteins called vitamin K dependent proteins (VKDP). VKDP have to go through this post-translational gammacarboxylation to be able to chelate calcium and activate their physiological properties (64–67). During the reaction, VitK_HQ_ is oxidized and oxygenated into vitamin K epoxide (VitK_OX_). Since dietary intake is often insufficient to meet the vitamin K need (66), VitK_OX_ has to be recycled in VitK_HQ_ to maintain the gamma-carboxylase activity. The recycling is performed by the vitamin K epoxide reductase enzyme (VKORC1) and it takes place in two stages (Figure 1): (1) a deoxygenation of VitK_OX_ catalyzed by the Vitamin K Epoxide Reductase (VKOR) enzyme leading to the formation of Vitamin K Quinone (VitK_Q_) and (2) a bielectronic reduction of VitK_Q_ in VitK_HQ_ mostly catalyzed by the VKORC1 enzyme and as an alternative minor pathway by the consensual NAD(P)H:Quinone Oxidoreductase 1 (NQO1) enzyme that is not the sole alternative reduction path (69–71).

Anticoagulant rodenticides are inhibitors of VKORC1 (72). When their concentration reaches a sufficient threshold, vitamin K recycling is stopped. Four clotting factors, the factor II, VII, IX, and X, synthesized by the liver, are among the VKDP. Consequently, under the effect of AR, these vitamin K dependent clotting factors are no longer activated by gamma-carboxylation and the blood concentrations of active clotting factors decrease leading to delayed uncontrol bleeding and sometimes death (64–67). This delay in action prevents the rodent to associate the effects with the ingestion of baits and explains the efficiency of these rodenticides. Moreover, in case of accidental exposure, the delay of action eases the implementation of a treatment.

SGAR molecules are little more potent than FGAR ones to inhibit VKORC1 of susceptible rat (73). Nevertheless, SGAR are more than 10 times more efficient than FGAR on resistant rodents (73). This potency to inhibit recycling mechanism of resistant rodent is one aspect of the effectiveness of SGAR. Moreover, it seems that this characteristic is shared among stereoisomers of a same molecule (74).

While vitamin K dependent clotting factors are the main VKDP affected by an AR exposure, long-term exposure may affect other VKDP like osteocalcin (OC) or matrix Gla protein (MGP). These proteins are involved in bone formation and energetic metabolism for osteocalcin (75, 76) and in the protection of soft tissue calcification for MGP (77).

#### General Pharmacokinetic Properties of Anticoagulant Rodenticides

AR molecules are rapidly and efficiently absorbed after ingestion (78). Moreover, some molecules can also go through the cutaneous barrier (79–81). The distribution of AR molecules through the organism differ widely between FGAR and SGAR. SGAR molecules are more liposoluble than FGAR and distribute more largely in hepatic tissue than other tissues like kidney and circulate slightly and transiently in plasma (82, 83). Conversely, while they are also found in liver, a significant amount of FGAR molecule circulates through the blood (78, 83). In addition, AR molecules can be excreted in milk and egg (84, 85). After an oral intake, the maximum liver concentration of the majority of AR molecule is reached in 4–8 h (66, 83, 86).

**Figure 2:**
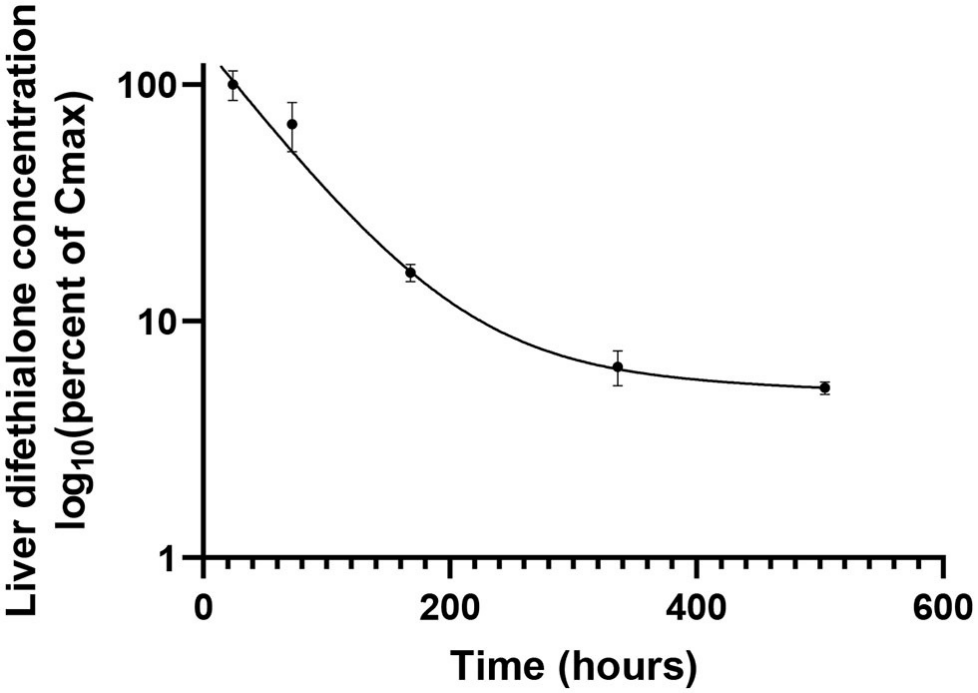
Liver concentration of difethialone over time expressed as percent of Cmax in rats after an oral doses of 3 mg/kg body weight. From data of Damin-Pernik et al. (62).

Table 1 presents hepatic half-lives of different AR molecules. Nevertheless, these data are to be taken with care. Indeed, these half-lives have been measured after a very low exposure and with a monitoring of only some weeks while half-lives reported are for some molecules of more than 100 days. Moreover, these results are inconsistent with other pharmacokinetic studies that report half-lives in hours (62). These discrepancies can be explained by a bi-phasic elimination with an initial- and a terminal-half-life. Figure 2 shows the hepatic pharmacokinetic of difethialone in rats. During the first decay phase the initial half-life is of 44 h while during the terminal phase the half-life is of 74 days like previous reported data in the table. However, when slow decay begins 95% of liver difethialone has been yet eliminated. Thus, final half-lives have to be interpreted with care. Nevertheless, the difference between the first and the second generation is clear and well-known, SGAR are more persistent than FGAR. This persistence explains, in part, the efficiency of SGAR. Indeed, while the baits including FGAR require several ingestions over several days to be effective, baits including SGAR require theoretically only one ingestion. Nevertheless, this advantage is also certainly responsible for SGAR high exposure and bioaccumulation in non-target species.

**Table 1:** Eliminated half-life for anticoagulant rodenticides in rat and mouse.

**Generation**	**Compound**	**Hepatic half-life (days)**	
		**Rat**	**Mouse**
1st generation	Chlorophacinone	35	35.4
	Diphacinone	3	–
	Coumatetralyl	55 - 62	15.8
	Warfarin	26.2–67	66.8
2nd generation	Brodifacoum	113.5–350	307.4
	Bromadiolone	170–318	28.1
	Difethialone	74–126	28.5
	Flocoumafen	215	93.8
	Difenacoum	118–128	61.8

*Modified from: (87–92)*.

As presented above, all stereoisomers of SGAR are not found in exposed non-target animals (56). This observation can be explained by the different persistence between stereoisomers. Indeed, while some stereoisomers are still quantified for many days after a lethal exposure to SGAR, others are not because they have a very short hepatic half-life (62, 74, 93). For example, the four stereoisomers of difethialone have initial half-life of, respectively, 6, 25.4, 69.3, and 82.3 h (74). Thus, only the last two stereoisomers contribute to the secondary exposure of non-target species.

AR are metabolized through cytochrome P450. Cytochromes involved in the AR degradation are different according to the nature of the molecule and its stereochemistry (94). These specific pathways for each stereoisomer could be the source of their different half-lives (95). If a part of some FGAR can be eliminated through urine (96, 97); the majority of AR are mainly or exclusively eliminated through feces (86, 87, 98, 99).

### Idiosyncratic Susceptibility of Individuals to Anticoagulant Rodenticides

Not all individuals respond equally to exposure to AR. According to their species, sex or genetic, animals will be more or less susceptible to AR. Table 2 presents LD50 of some AR according to the species exposed. This table pinpoints the wide discrepancy between species considering AR susceptibility. Moreover, some species are more susceptible to some molecules without a correlation with their generation. For instance, pigs tolerate difethialone more than brodifacoum or warfarin (106). In this part, we focus on the reasons of these differences.

**Table 2:** Single-dose oral LD50 (mg/kg body weight) in different animals.

	**Chlorophacinone**	**Diphacinone**	**Difenacoum**	**Brodifacoum**	**Difethialone**	**Bromadiolone**
Rat	2–20.5	0.3–30	1.8	0.27–0.5	0.56	0.56–1.12
Mouse	1–20.5	141–340	0.8	0.4	1.29	0.99–1.75
Guinea pig	–	–	50	0.28	–	2.8
Rabbit	50	35	2	0.2–0.3	5.3	1
Dog	–	0.88–15	50	0.25–1.0	4	8.1–10
Cat	–	5–15	100	0.25–25	–	25
Pig	–	150	80	10	2–3	3
Chicken	–	–	50	3.15–20	–	5
Sheep	–	–	100	10	–	–
Mallard duck	100	3,158	2	4.6	–	–
Wild birds	430	–	–	–	–	–
Quails	–	–	–	–	–	1,600

*Modified from: (10, 100–105)*.

#### Variations of Susceptibility to Anticoagulant Rodenticides Related to Pharmacodynamics

**Figure 3:**
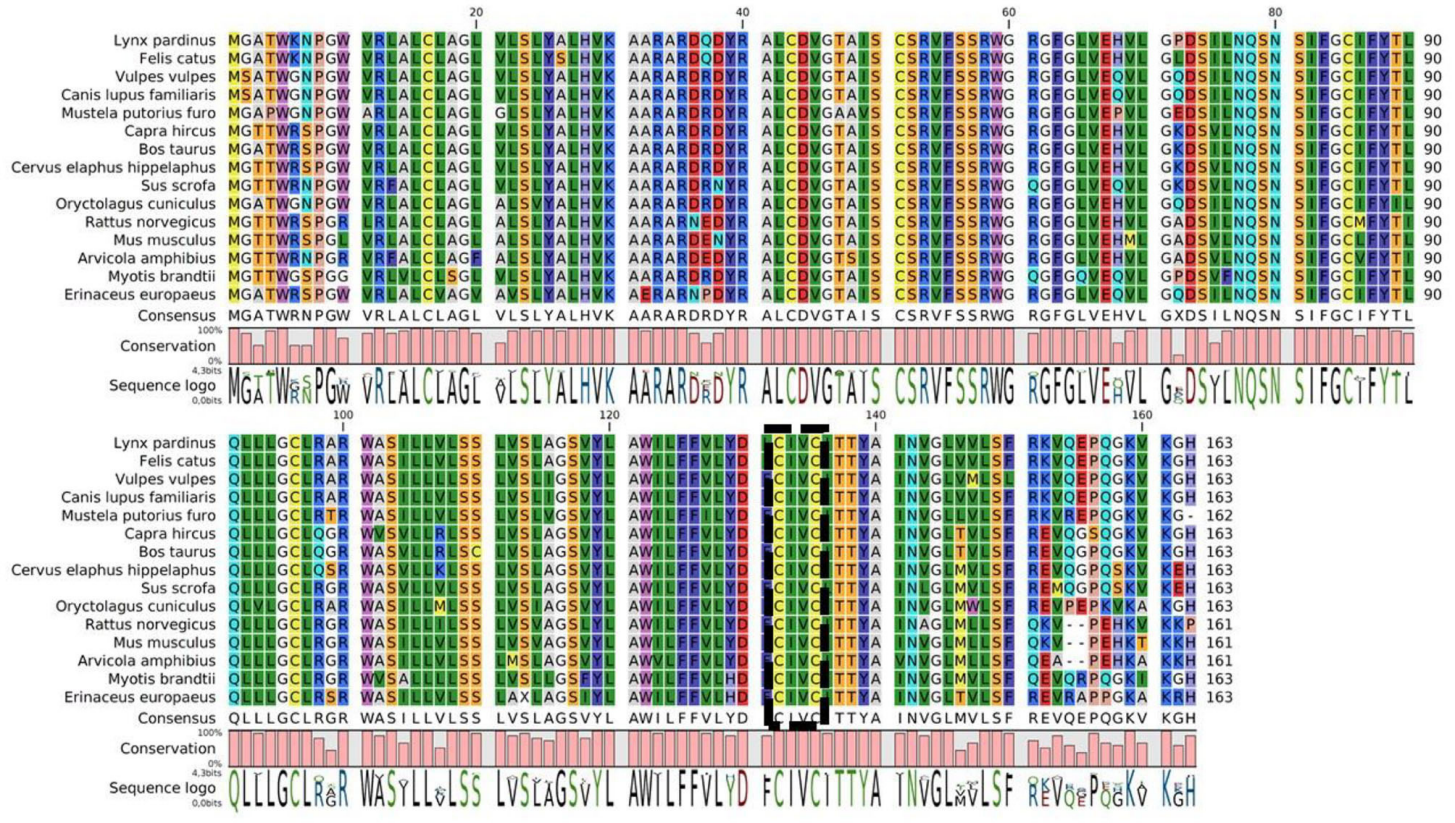
Multiple sequence alignment of VKORC1 enzymes of some animals who might be exposed to AR. The active site is indicated by the box.

A large part of the differences of susceptibility to AR might be explained by a difference of VKORC1 sequence among species. Figure 3 shows a multiple sequences alignment of the VKORC1 amino acids. While the active site is well-conserved between species, there are multiple changes among other parts of the protein. The sequence identity (conserved amino acids) between the 15 sequences presented is only of 60% and their homology (conserved amino acids and amino acids with similar chemical properties) is of 83%. The change of only one amino acid may induce a major resistance to AR of the VKORC1 enzyme. Indeed, VKORC1 amino acid changes have been demonstrated to be involved in the difference of susceptibilities among same species, as described in mice, rat and human (23, 73, 107, 108) and the intensive use of AR has selected the most resistant variants of VKORC1 among rodent populations (23, 73). However, this difference of susceptibility to AR differs according to the mutation and to the AR considered (73). For many species, VKORC1 polymorphisms and their consequences on the susceptibility to AR are unknown.

Another possible source that may explain differences in sensitivity is the vitamin K dependent clotting factors pool and its rate of decrease after the inhibition of VKORC1 by AR. This mechanism is proved in the difference of susceptibility between female and male rats (66). Indeed, females have higher basal pool of factor VII and the decrease of plasmatic factor II and factor X are slower than males. Moreover, there is a lag time of 4 h before factor VII begins to decline in females. Thus, female rat coagulation is slower impaired by AR than male one (66). The difference of susceptibility according to the sex differs among species. For instance, female mice resist less to AR than males.

Finally, food could be a pathway of resistance. Indeed, a vitamin K rich diet might reduce the action and the efficiency of AR exposure by providing the antidotes to the animals. However, this kind of resistance has not been clearly demonstrated or identified in wildlife populations (109, 110).

#### Variations of Susceptibility to Anticoagulant Rodenticides Related to Pharmacokinetics

Pharmacokinetics may be another source of susceptibility or resistance to AR. As discussed previously, the hepatic half-lives of rodenticides differ widely between rats and mice. Moreover, there is no general rule to convert rat half-life to mice one, pharmacokinetic parameters should be determined for each pair molecule-specie. Moreover, some bird species like *Gallus domesticus* showed greater metabolic activity of warfarin compared to rat whereas very little warfarin metabolism was demonstrated in owls which can explain the recurrence of reported poisoning in these species (111). In addition, compared to red deer and pigs, cattle has different pattern of distribution and metabolization of diphacinone with rapid initial phase of decline and longer terminal hepatic half-life suggesting a more developed binding of diphacinone in the liver of cattle or advanced enterohepatic circulation (112). More generally, since expression of cytochrome P450 and xenobiotic metabolism pathways differ among species (113), it is not possible to generalize the elimination rate of AR for all species. This elimination rate defines the persistence of the molecule in non-target species and therefore its ability to bioaccumulate.

Among a same species, some lines of animals have a better metabolism of AR that may lead them to resist to some rodenticides. It is the case of a line of roof rat (*Rattus rattus*) (114) in Japan, of the Berkshire line of brown rats (*Rattus norvegicus*) (115) and of a population of California ground squirrels (*Otospermophilus beecheyi*) (116). The overexpression of P450 cytochromes involved in AR metabolism was often undefined or suspected. As these expressions are also sex dependent, it could be linked to the AR susceptibility difference between male and female. Indeed, in many species P450 cytochromes are overexpressed in female (116, 117). Despite that pharmacokinetics are not always associated with susceptibility or resistance, in Lefebvre et al. (66) liver difethialone area under the curve and maximum concentration are almost twice higher in female rats than males, yet females are less susceptible than males.

### Anticoagulant Rodenticides Exposure, When to Suspect It?

Considering the extent of AR exposure in domestic and wildlife fauna, it is essential to monitor it. The first clues of an animal exposure are clinical signs suggesting of an acute exposure. However, the consequences of chronic exposure, even if less known, as well as animals that are asymptomatic but at a high risk of exposure must also be considered in AR exposure monitoring.

#### Acute Clinical Signs That Are Suggestive but Not Specific

Clinical signs of rodenticide intoxication are quite similar for higher vertebrate species: hemorrhagic syndrome, with main localizations being respiratory (coughing/hemoptysis, nasal bleeding, pulmonary oedema, intrapulmonary, and pleural hemorrhages) and digestive (hematemesis, hematochezia, and melena) tracts, and less specific symptoms including lethargy, anorexia, pallor or ataxia, that are usually the first signs to be noticed (118–122). Clinical onset is usually delayed from 2 to 10 days post-ingestion (36, 121).

Numerous case reports in dogs provide extensive descriptions of the clinical signs observed. Apart from general and non-specific signs, and respiratory and digestive bleeding disorders, tachycardia and dyspnea with polypnea are reported, as well petechiae and ecchymoses on the skin and mucosa, and pallor (11, 119). Some reports mention also subconjunctival hemorrhages (123). At a later stage of the intoxication (after 1 week), a bilateral symmetrical enlargement of the abdomen can also be observed in certain cases, frequently accompanied by prolapse of the ventral abdominal wall and lordosis of the vertebral column, resulting from hemorrhagic effusion (ascites). The formation of massive hematomas after venipuncture is also quite characteristic (11).

Ruminants are apparently less often exposed than other domestic species (only 2.2% of calls to the CNITV about rodenticide poisoning are for ruminants), and display clinical signs less frequently (only 10.5% of exposed animals displaying clinical signs according to CNITV data—without further details on the severity of those signs) (36). Case reports in ruminants (cattle, sheep) mention that overt clinical signs of intoxication are usually absent (85, 124), with only a slight increase of bleeding time at the venipuncture site in some cases (125). This decreased incidence of clinical signs is possibly due to a lower sensitivity to AR, that could be related to a dilution effect in the rumen, endogenous production of vitamin K1 in the rumen, or dietary vitamin K in their feed composed of leafy greens (85, 125). Still, AR intoxication might be more severe in young animals, as one case report mentions fatal hemorrhages in lambs following exposure to chlorophacinone (126). Reports on horses are less frequent, but still some cases of AR intoxication are described, with similar symptoms to other species (127, 128). In one case, AR toxicosis is suspected to have led to the abortion of the mare (129).

AR toxicosis in wild animals, especially birds of prey, is also quite well-described in literature. Similar signs are reported, observed ante- or post-mortem, with a great focus on overt hemorrhagic lesions (e.g., bruising, bleeding from orifices, hemorrhages of cavities, and gastro-intestinal tract) (130, 131). In some cases, there might be no frank evidence of bleeding, and microscopic hemorrhages in tissues (heart, liver, kidney, lung, intestine, skeletal muscle) should be sought by histological examination to confirm diagnosis (132).

In laboratory rats, transient early hemoglobinuria (associated with oxidative stress) with consecutive late hematuria (associated with anticoagulation) were observed after poisoning with brodifacoum, that could be used as novel clinical biomarkers of AR poisoning, if generalizable to other species (133). Moreover, in human treated with warfarin soft vascular calcification may occur (134). This adverse effect of anti-vitamin K anticoagulant has been confirmed in rat model (135). Nevertheless, to the knowledge of the authors, such calcifications have not been suited in AR exposed fauna but would deserve to be studied.

Thus, hemorrhage are important markers of acute AR toxicity in higher vertebrate species. Moreover, they can be used in both alive and dead animals. Nevertheless, hemorrhages are not specific of AR intoxication, as they might be the consequence of trauma (consecutive to AR intoxication or not) or diseases like leptospirosis (136) or other causes of bleeding. Thus, if the presence of hemorrhages is an important clue in favor of AR poisoning, it is not sufficient to conclude.

When hemorrhages occur in living animals, the therapeutic response to vitamin K injection may allow to reinforce or reject an AR intoxication hypothesis. However, false positive occurs in the case of vitamin K deficiency (due to an unbalance diet) or of genetic VKORC1 activity deficiency (137). However, in the case of an exposure of a large and or wild animal the cost of the treatment may raise the question of its necessity. Is the exposure sufficient to threat the animal health and when should we stop the treatment? Indeed, because of the persistence of SGAR, treatment lasts at least 5 or 6 weeks, and in human medicine its courses averaged 168 days, is it sufficient or too long? The current method to set the duration of treatment is, to stop it after 1 month of treatment for 48 h then to assess the prothrombin time (PT) (36). Is there a biomarker that can predict if the treatment is necessary and when to stop it without discontinuing the treatment?

#### Clinical Signs Suggestive of a Chronic Exposure to Anticoagulant Rodenticides

Clinical signs of a chronic exposure are currently not sufficiently characterized to be used to suspect an AR exposure at the individual scale. More and more studies suggest that the effects of AR are not confined to coagulation alone. Indeed, the activity of VKDP unrelated to coagulation may also been impacted by AR. More precisely the bone metabolism may be influenced by the decrease of osteocalcin carboxylation and vascular calcification prevention by that of MGP. Decrease in bone density and increased risk of fractures (by under-carboxylation of osteocalcin), have been investigated in AR-exposed predatory birds, without any conclusive result (138). However, studies based on rodent model show teratogenic activities of some AR with consequences on bone formations (139, 140). Conversely, the relationship between chronic exposure to warfarin and valvular and coronary calcification has been widely described in human (as part of anticoagulant treatment) and in rodent model (135, 141). To the author knowledge vascular calcification have not been studied in exposed wildlife population.

Beyond effect related to VKDP, AR are suspected to increase susceptibility of bobcats (*Lynx rufus*) to notoedric mange, with some fatal outcomes, by interfering with the immune system of the exposed animals (142, 142). The effect of AR on immunity has been confirmed in rats and on human cells (81, 143, 144). Another studies show that chronic exposure could have an effect on genetic expression of bobcat (145). In addition, as described in rodent, AR might be a source of behavior modification (33–35). Thus, the consequences of chronic exposure on the health of non-target animals could be very diverse and should be better studied. Indeed, with this lack of characterization it may be difficult to distinguish the effect of AR and non-AR molecules in the case of multiple contaminations.

As the clinical signs of a chronic exposure are not well-established, AR exposure should be suspected at animal population level. The sign of appeal might be, bone deformation or abnormalities (notably in new-born), unusual infectious susceptibility for no obvious reason (immunodeficiency virus…) and vascular calcification at necropsy. Moreover, chronic exposure with only slight clinical signs may not be identified, the criteria for suspicion of asymptomatic exposure are also relevant.

#### Asymptomatic Exposure to Anticoagulant Rodenticides

Due to the difference of susceptibilities of animal species to AR, to the level of exposure or to the delay after exposure (few hours to few days), some animals may not present any clinical sign whereas they have been exposed to AR. If it is not a problem for the animal itself; it can be, depending on the situation, for other individuals, for animals in the food chain or even for humans. This animal can be a source for secondary AR exposure. Indeed, this animal or its products can be consumed.

In the case of domestic animals, as exposures are mainly primary and occurs in a controlled environment, asymptomatic exposure to AR is suspected when animals were able to get access to the bait. There are few studies on the risk of the consumption of products from exposed animal. However, as AR can be excreted in milk and eggs (84, 85), it is advisable to withdraw these products from human and animal consumption and to contact a veterinary poison control center.

Considering wildlife, it is more complicated as it is not possible to suspect if the animals have been exposed (no controlled environment). It is an issue for the wildlife, as exposed animal can be a source of secondary exposure for predator. Thus, it is a major concern for hunted species like wild boar which can be a source of human exposure. According to the exposure studies on small mammals, predators and wild boar, wild animals should be suspected of being exposed when they are found close to areas with significant human activity (urban area, farms…) (44, 46, 49, 51). Moreover, in some countries, they can be both a pest (and treated with AR) and a food source (146). This makes it necessary to provide pedagogical support to the population and a risk-benefit approach to the use of AR.

In the presence or absence of clinical signs, a suspicion of exposure to AR should be confirmed and its biological consequences assessed, in order to measure the impact of these molecules on non-target species. This monitoring will make it possible to measure the effectiveness and relevance of new practices and methods for controlling rodent populations with a view to reducing the exposure of non-target populations.

## Biomarkers for Anticoagulant Rodenticides Exposure Assessment

### Definition and Efficiency Criteria

Biomarkers are measurable parameters at biochemical, histological, immunological, physiological, or organismic levels, that are used as indicators of certain biological states or conditions (147). They are relevant in many scientific fields, especially in ecotoxicology, but also in the biomedical field.

In the field of ecotoxicology, there is a constant need to detect and assess the impact of pollution, particularly in cases of low concentrations of increasingly complex mixtures of contaminants. This has led to an increasing number of studies on the development of indicators of the biological effects of contaminants on organisms.

A common dichotomy for biomarkers in ecotoxicology is between biomarkers of exposure, and biomarkers of effect. While biomarkers of exposure are relevant for hazard identification (e.g., for xenobiotics, identification of the parent compound, or derived metabolites), biomarkers of effect are used for hazard assessment, as they assess the response of the exposed organism to the particular xenobiotic or complex mixture (147). A third category would be biomarkers of susceptibility, used for indicating the inherent or acquired ability of an organism to respond to the challenge of exposure to a specific xenobiotic substance.

Biomarkers are sought at different levels of the organisms (148, 149). They may be inducible enzymes playing a role in the xenobiotics elimination (i.e., phase I biotransformation enzymes—such as isoforms of cytochrome P450—and phase II enzymes such as gluthation-S-transferase), but also oxidative stress parameters, biotransformation products (e.g., metabolites levels in body fluids), stress proteins (heat-shock proteins), hematological parameters (e.g., delta-aminolevulinic acid dehydratase—ALAD—as a biomarker applicable to lead exposure), immunological parameters, reproductive and endocrine parameters, neuromuscular parameters (e.g., acetyl cholinesterase inhibition by organophosphorus and carbamate agricultural pesticides), genotoxic parameters (e.g., DNA adducts), physiological and morphological parameters (e.g., asymmetric development, organ size variations), or even proteomics and genomics, which provide an integrated picture of the way an organism responds to a changing environment (150).

It is worth noting that misapplication or misinterpretation of biomarkers in ecotoxicology may lead to erroneous conclusions. The first potential biases arise from handling conditions of samples, including their collection and storage that might affect the final quantitative and qualitative assessment of the biomarker. Sample relevance may decrease due to biological (decomposition process including other living organisms) and physicochemical (oxidation, denaturation, hydrolysis…) factors that cause potential instability of the biomarkers. In general, ante-mortem samples are better processed because they are taken fresh from a live animal. This does not apply to those taken from animals found in the wild without knowing the time of their death therefore ignoring the post-mortem interval before their collection. Carcasses should ideally be collected upon animal death and specimens should be fixed in liquid nitrogen transported and stored at −80°C to ensure biomarker preservation. For an accurate interpretation of circumstantial changes, a thorough understanding of the post-mortem process of the biomarker in different organs regarding to its constancy over time and environmental factors (temperature, photoperiod, etc.) is necessary.

Interspecies extrapolation is not always possible, as well as translation from the laboratory to the field situation, and the impact of non-pollution-related variables (health condition, gender, age, nutritional status, metabolic activity, migratory behavior, reproductive and developmental status, population density, season, ambient temperature, heterogeneity of environmental pollution) should be considered as confounding factors (148). The relevance of the use of biomarkers is therefore sometimes questioned (151), as they may be useful as indicators of exposure and for the formulation of hypothesis in carefully controlled experiments, but might not be expected to provide useful predictions of relevant ecological effects, which does not meet the aims of environmental monitoring and ecological risk assessment to detect and/or predict adverse chemical effects on populations, communities, and ecosystems. Still, those markers may be relevant for long-lived species, or rarer species, for which non-invasive indicators are needed.

For non-target fauna poisonings by anticoagulant rodenticides (AR), while clinical biomarkers are relevant for the identification of individual poisonings in domestic and wild animals, ecotoxicological biomarkers are needed to assess the extent of exposure to AR and the sublethal effects of these contaminants on wildlife populations. Based on the criteria that have been defined to identify relevant biomarkers in the environmental risk assessment (ERA) for chemicals (149), we propose seven criteria for assessing the relevance and condition of use of AR biomarkers:

- Development stage: is the biomarker only an idea, or used for research purposes or sufficiently standardized to be used on the field?
- Dead or alive: does the biomarker is usable for a dead animal, a living animal or both?
- The availability of usual values or thresholds for the considered species.
- Compatibility with treatment with vitamin K: can biomarker be used to assess AR exposure or potential effects during treatment?
- Sensitivity: does biomarker can detect limited exposure to AR or confirm a symptomatic exposure?
- Specificity: are there confounding factors for the biomarker?
- The toxicological significance of the biomarker (relationship between response and impact to the organism) must be demonstrated.

Table 3 gathers and assesses the main biomarkers identified in this review according to previous criteria.

**Table 3:** Biomarker assessing the exposure and the effect of anticoagulant rodenticides in animals.

**Biomarker**	**Tissue**	**What is dosed**	**Dosing methods**	**Development stage (experimental, research, available)**	**Dead or alive**	**Stability of sample over time**	**Available usual value**	**Compatibility with treatment**	**Sensitivity**	**Specificity**	**Toxicological significance**
AR dosing	Liver (organs)	Levels of AR in the tissue—detection can be stereoisomer-specific	Usually LC-MS/MS	Available	Dead (or through biopsy)	Really stable	NA	Yes	High	High	Proposed threshold for birds of prey, to be further investigated
	Plasma				Alive		NA	Yes	Low	High	No
	Feces				Alive or freshly dead	Quick decrease over time when exposed to weathering	NA	Yes	Low	High	No
Prothrombin time	Plasma on citrated tube	Ratio based on coagulation time of a citrated plasma, without platelets, with calcium and thromboplastin	PT assay	Available	Alive	NA	Available for domestic animals	No	Medium	Medium	High in acute exposure
Vitamin K dependent clotting factors	Plasma on citrated tube	Activity of clotting factor	Chromogenic assay	Research	Alive	NA	No in animals	No	Medium	High	High in acute exposure
ucOC	Plasma	Undercarboxylated osteocalcin as the percent of the total osteocalcin in the sample	Radioimmunoassay (RIA) or ELISA	Experimental	Alive	NA	NA	Unknown	Unknown	Unknown	Unknown
ucMGP	Plasma and some tissues	Undercarboxylated, inactive species of MGP	Radioimmunoassay (RIA) or ELISA	Experimental	Both depending on the tissue considered	NA	NA	Unknown	Unknown	Unknown	Unknown
VKOR activity from liver extract	Liver	Ability to produce vitamin K quinone from epoxide form	Enzymatic assay	Research	Dead	Has to be immediately store at −80°C	NA	NA	NA	NA	Assess one mechanism of resistance
VKOR activity from VKORC1 expressed in yeast or cell	DNA sample or VKORC1 sequences	Enzymatic assay : Ability to produce vitamin K quinone from epoxide form Cell assay : ability to produce carboxylated proteins	Enzymatic or cell assay	Research	Alive	Long	NA	NA	NA	NA	Assess one mechanism of resistance
Vitamer quantification	Liver, kidney or lung	Vitamin K1, and MK-4, in quinone and epoxide forms	HPLC-fluorescence	Experimental	Dead	Fairly stable at room temperature	Epoxide forms are absent in healthy animal	Yes	Unknown	Quite high (author data)	High in acute exposure, unknown in chronic exposure

*NA, not available*.

### Exposure Biomarkers: the Concentrations of Anticoagulant Rodenticides Molecules

Non-invasive detection and quantification of AR can be performed in plasma (85, 152–155), milk (85, 124), and feces (37, 154, 156, 157), while invasive analysis can also detect AR in target organs, especially in the liver, that is widely used for forensics investigation especially for wildlife (61, 130, 131, 158–160), but also in muscle, fat, or bone (155). Analyses are usually performed by high performance liquid chromatography that can be coupled with UV, fluorescence (152) or mass spectrometry (157, 160–162) detection.

AR screenings can be relevant to confirm and quantify exposure to these chemicals. For domestic animals, it can be a valuable tool for confirming the diagnosis of AR intoxication in cases where ingestion has not been observed, (152) but it is not always really relevant, as blood parameters are quite sufficient to determine the need of treatment, and as it is not a possible routine exam in clinical settings. Furthermore, severity of clinical signs or extent of PT prolongation do not seem to be correlated to AR concentration in dogs (152) or sheep (85).

As feces are the main route of AR elimination, their use to monitor exposure to AR has been widely investigated, both for domestic species (e.g., sheep, dogs) (154, 155) and wildlife, with investigations on foxes (157, 163). Further research is still needed on the elimination pattern for each AR, to accurately monitor exposure to those products, and some limits for this method to be used on the field is that AR are not stable in feces when outdoor, so only fresh samples (<5 days) should be used for monitoring (163). Moreover, plasma and fecal levels of AR are not correlated with liver levels (37, 154, 164). Some AR such as bromadiolone circulate poorly in the blood. Thus, the assessment of exposure through plasma or fecal material may lead to false negative results or underestimated exposure.

Investigations on AR levels in the liver of necropsied birds of preys have been widely conducted, with the purpose to determine a threshold that could allow to differentiate between simple environmental exposure and real intoxication on those animals, and thus to better assess the ecological impact of AR on the wildlife population. A repeatedly cited toxicity threshold is above 100–200 ng/g wet weight (165), but was determined rather as a potential lethal range, and derived from a single species, the barn owl (*Tyto alba*). More recent research has been conducted by combining both published data and results from surveillance programs in Canada and using logistic regression to estimate the probability of toxicosis associated with different liver AR residues. It described a significant likelihood of toxicosis in 5% of individuals with liver concentrations of 20 ng/g wet weight and in 20% of individuals with concentrations of 80 ng/g wet weight (160). Still, one of the limitations pointed out in the study, as well as in some other publications (159), is the species-specific sensitivity that prevents any extrapolation of a potential threshold, especially as a discrepancy exists between birds tested in captivity which might be more tolerant to AR than free-ranging birds with more complex daily activities and environmental stressors. Some authors thus consider that such a parameter may not be a relevant diagnostic tool, as it is neither accounting for the impacts of sublethal effects on reproduction and non-target mortality, nor for bioaccumulation of repeated sublethal exposures (166). Indeed, it has been demonstrated that subsequent exposures to AR may lead to increased adverse effects on the contaminated birds (159). Finally, it has also been suggested that the use of total sum of AR to assess the extent of exposure should rely on a quantification expressed in nmol/g instead of ng/g, that would better reflect the inhibitory potency on VKOR for each AR (159).

Beyond the assessment of exposure, liver AR dosing and more specifically the dosing of stereoisomers may provide information on the type of exposure and in the case of primary exposure may help to estimate the time from bait ingestion to death. Because stereoisomers have different elimination rates, the longer the time between ingestion and death, the greater the proportion of persistent enantiomers in the total residue (62, 74, 93). Conversely, the shorter the delay, the closer the ratio of enantiomers will be to the initial bait. This clue on time between ingestion and death might be useful to determine the cause of death. Indeed, it is unlikely that an AR can kill in <2 days. Moreover, a ratio of stereoisomers close, to that of the bait is in favor of a primary exposure.

### Effect Biomarkers: Highlight Anticoagulant Rodenticides Effects

#### Blood Clotting Assessment

As anticoagulant rodenticides main action is on coagulation cascade, blood clotting assays are obvious and relevant diagnostic tools.

Prothrombin time (PT) measured on citrated plasma samples is considered a sensitive test to confirm AR contamination, as it is the first coagulation parameter to be altered after AR exposure, due to the short half-life of factor VII (6.2 h) (167). It should be noted, however, that in animals with clinical bleeding both PT and aPTT (activated Partial Thromboplastin Time) are prolonged, while thrombin time remains normal. Still, since assesses the extrinsic—or tissue factor—pathway of coagulation, it remains a more specific marker of AR intoxication, and is considered as a clinical standard for the diagnosis of AR intoxication (36, 120, 121, 152, 168).

PT as a diagnostic tool for AR contamination is widely described in dogs (118, 119, 121–123, 152, 168). Basal value for dogs is around 6–10 s, and is greatly prolonged after AR exposure, with reported median values between 40 (119) and 52.3 s (152). A commonly used threshold is a 25% increase over a control animal.

In ruminants, same mechanisms are expected, and there are also some reports of increased PT after AR exposure in cattle (124), sheep (85, 125), and goats (169), with similar variations. In one study, baseline PT for sheep was around 13.25 s (SD = 0.52), increasing up to 27–40.7 s after administration of the AR (125). As reference baseline values cannot be easily found for those animals, and values may differ among breeds and the analytical method used (170) [e.g., for sheep median values vary from 7.31 s in “mixed breed” ewes (171) to 40 s in a study with Austrian Mountain ewes (172)], the use of a control animal may be beneficial (85).

While PT seems a good biomarker of AR intoxication in domestic animals, no linear correlation was found between AR concentrations and extent of PT prolongation neither in dogs (152) nor in sheep (85).

It is worth noting that some research has previously been performed on the use of Proteins Induced by Vitamin K Antagonism or Absence (PIVKA) for the sensitive diagnosis of AR intoxication in dogs (173). The PIVKA test was originally designed to monitor human patients under treatment with coumadin and is still use in human medicine for diagnosis of e.g., hepatocellular carcinoma, pancreatic, or biliary tumors. This test is quite similar to the PT-test, using a diluted plasma sample and an “altered” thromboplastin that delays the *in vitro* clot formation, and thus may be more sensitive in detecting prolongation of clotting time by AR. Still, as the PT variation after AR exposure is usually marked, and with the availability and standardization of PT reagents, the PIVKA test was quite abandoned as a diagnostic tool, especially as results of this test can also be increased by deficiencies in factors II, VII, and X because of heredity and acquired coagulopathies other than anticoagulant poisonings, and is sensitive to the presence of heparin (168, 173).

Blood assays are also used for diagnosis of AR exposure in wildlife animals, especially birds of prey (61, 132, 174–176). In those animals, lengthening of PT by more than 25% or two standard deviations above baseline values is considered as suggestive of AR exposure (61). Although blood clotting assays seem promising for diagnosis of AR exposure in wildlife, there is still a need to establish species specific reference values and standardize assay methods among testing facilities (175). Among observations in wild birds, some studies showed that far greater PT values were observed in birds undergoing subsequent challenge exposure to AR than the ones observed in previously unexposed birds, suggesting a potentiation of AR adverse effects after repeated exposure as a sub-lethal effect of AR (158, 159). One limit of the PT assay in birds is the recommended use of homologous (i.e., avian) thromboplastin, not readily available, to efficiently trigger *in vitro* coagulation. Great variations (up to three-fold increase of PT, and even higher increase for aPTT) can occur when using human or mammalian (rabbit, bovine) thromboplastin for avian clotting assays (177–179). To complete avian blood coagulation assays, another test, Russel's Viper Venom Time, is routinely used, (159, 174–176), with observed increases of both “adapted” PT time and Russel's Viper Venom Time within 48–96 h following the exposure to AR (174). Russell's viper venom is known to activate factor X in the common pathway of the clotting cascade (180). Finally, thrombin generation test and thromboelastometry can also be used, even if they don't bring any added value (181, 182).

#### Vitamin K-Dependent Proteins (VKDP) Dosage

Vitamin K-dependent proteins (VKDP) dosing might be an important source of biomarkers as the inhibition of the recycling mechanism of vitamin K has direct consequences on those proteins. Nevertheless, the challenge is not only to dose VKDP but also to dose separately carboxylated and under-carboxylated VKDP (ucVKDP). Indeed, AR molecules act on the post-translational gamma-carboxylation mechanism but not on the genetic expression of VKDP.

##### Vitamin K-dependent clotting factors

The dosing of vitamin K dependent clotting factors might be a good indicator of AR intoxication. These dosages are based either (i) on the evaluation of the activity of the clotting factor under consideration through a reaction cascade (close to the coagulation cascade) leading to the production of a chromogenic factor and where the dosed factor is the limiting factor, or (ii) on the supplementation of plasma depleted in the factor considered. Only carboxylated clotting factors should be assessed. As carboxylated clotting factors decrease faster than increase in clotting times arises, their dosing allows early detection of intoxication. For example, in rats and after an ingestion of a lethal dose of AR; the factor VII and factor X activity are halved in about 4 and 5 h, respectively, while prothrombin time doubles in 10 h (182). However, as the assessed clotting factors have to interact with reagent component optimized for humans, it is not obvious that this method is conceivable for all species. Moreover, there are not usual values for animals.

##### Other vitamin k-dependent proteins

If vitamin K dependent clotting factors are linked to the acute toxicosis of AR, other VKDP might be linked to their chronic effects. The two major non-clotting VKDP are osteocalcin (OC) and Matrix gla protein (MGP).

Osteocalcin limits bone formation without impairing bone resorption or mineralization (76). Under-carboxylated osteocalcin circulates naturally in bloodstream and seems to have positive effects on insulin sensitivity (183, 184). Moreover, several factors influence serum under-carboxylated osteocalcin levels notably osteoblastic synthesis, hormonal status, renal function, age, sex, timing of blood sampling, and specificity of the radioimmunoassay (185–187). Recently, a study showed that under-carboxylated osteocalcin increases in resistant rodents under vitamin K deficient diet (188). But currently there is no link between osteocalcin and chronic AR exposure effects. So, further research is needed to determine if carboxylated osteocalcin or under-carboxylated osteocalcin can be useful as biomarker of AR effects.

Conversely, under-carboxylated MGP (ucMGP) is well-known to be in favor of vascular calcification (189, 190), which is one of the suspected effects of chronic exposure to AR. In rats an increase of ucMGP resulting from a vitamin K deficiency is associated to vascular calcification (191). Further studies are needed to confirm this association and the link with chronic AR exposure, but circulating ucMGP might be a good candidate as biomarker.

MGP and OC are small proteins currently measured by immunoassays. Their sequences are variable between species, which raises the question of assay methods. Their assay will require the development of specific antibodies in the absence of methods to evaluate their activity. Moreover, as they are proteins, their stability at room temperature in animal cadavers will be a challenge. These biomarkers will eventually be used as research tools, but their use in surveillance studies seems difficult in the future. On the other hand, they could be dosed on live animals as well as on dead animals, from blood (OC and MGP), bone (OC), vascular, pulmonary and renal tissues (MGP).

#### VKOR Activity

In mammals, two different enzymes possess Vitamin K epOxide Reductase activity (VKOR) and are coded by paralogous genes. The enzyme called VKORC1 is mainly located in the liver and is the main contributor to the recycling of Vitamin K needed for activation of clotting factors, while the enzyme called VKORC1L1 does not sustain coagulation but maintain the Vitamin K cycle for carboxylation of other VKDP (192, 193).

In human, sensibility to warfarin before an anticoagulant treatment can be assessed by testing alleles coding for VKORC1 to detect the presence of potential variants susceptible to adapt the proper dose of warfarin (194, 195). These are consequently biomarkers of the susceptibility to a rodenticide and this pharmacogenomic approach could be used as a preventive measure.

Thus, the study of the differences in VKOR activity for each species can be useful to evaluate their sensibility to AR. This assessment is currently based on enzymatic assays (73, 108), that implies either to sample fresh liver from considered species or to use heterologous expression of VKORC1 in yeast or eukaryotic cells. Then the inhibition capacity of each AR molecule can be tested *in vitro* against VKORC1 of each species. Two methods are currently described to measure VKOR activity, the DTT-driven VKOR assay (73) and the cell-based VKOR assay (196). The first method allows the characterization of the susceptibilities of VKOR activity from animal tissues or recombinant VKORC1 enzymes. The second method allows the characterization of VKOR activity only from overexpression of VKORC1 enzymes and required thus to know the amino acid sequence of the VKORC1. Unfortunately, VKORC1 sequence is known only for few species. Nevertheless, characterization of VKOR activity by *in vitro* assays assesses only the susceptibilities linked to VKORC1 and is cumbersome to implement.

#### Vitamers Quantification: Picture of the VKOR Activity

Vitamin K is available in organisms under different forms, called vitamers. A distinction can be made between vitamin K1, also called phylloquinone, of vegetal origin, and menaquinones (MK), from bacterial origin. All those vitamers share a common 2-methyl-1,4-naphtoquinone cycle, corresponding to vitamin K3, derived from synthesis or catabolism. Vitamin K1 has a phytyl side chain at the 3-position, while MKs have several repeating prenyl units, the number of units being given as a suffix (menaquinone-n or MK-n) (197). Menaquinone-4 seems to have a major but not fully elucidated physiological role, and is the main form of vitamin K in the organism. It can be used in humans as drugs or supplements for newborns (198). MK-4 can be synthesized in the body, from vitamin K1 of feed origin or from vitamin K3, by a biosynthetic enzyme localized in the endoplasmic reticulum and ubiquitously expressed, known as UBIAD1 (199). MK-4 is also from microflora origin. Vitamin K1 is mainly found in liver and heart, with high storage capacities, while MK-4 is preferentially found in extra-hepatic tissues, especially pancreas and testis (198). Nevertheless, from the authors' experience, some species have hepatic MK-4 concentrations higher than K1.

VKORC1 is partly responsible for the recycling of vitamin K: when the VKOR activity is impaired by AR, it can be assumed that it has an impact on the concentrations of those vitamers, with a possible shift between epoxide forms and quinone forms. A dose-response relationship was suggested between ratio of epoxide and quinone forms of vitamin K1 in plasma and concentrations of two AR, phenprocoumon and warfarin (200). It was also proposed to used measurements of this ratio for diagnosis of AR intoxication in dogs (201). Moreover, during a treatment with vitamin K1, if an animal is intoxicated by rodenticides the epoxide forms will increase the same way as the quinone form, while if it is not, only the concentration of vitamin K quinone will be increased. Thus, this method could be used to assess an intoxication while under treatment. Further studies will be necessary to explore vitamin K and vitamin K epoxide concentrations as potential markers of AR exposure. Nevertheless, because differences in vitamin K concentrations between species are predictable due to differences in diet and microflora, determination of usual values would be necessary for each species. One way to overcome this difficulty may be to assess the ratio between quinone and epoxide forms.

The measurement of vitamin K forms is usually performed by chromatography (liquid, high performance), that can be coupled with fluorescence (152) or mass spectrometry (157, 160–162) detection. The determination of vitamin K concentrations can be done in plasma, as the different forms circulate in the plasma in live animals. Nevertheless, their concentration in the plasma could be biased by the last meal. The determination of vitamins K can also be done post-mortem from different tissues, liver, kidney, etc. Nevertheless, the relevance of this assay in different tissues in relation to AR concentrations will require further studies. Nevertheless, vitamin K concentration measurement appear to be a promising route to provide relevant biomarkers. Vitamin K forms appear to be rather stable when the samples are protected from light.

### Toward New Tools to Develop Biomarkers for Sublethal Effects of AR?

#### Cytochrome P450 Pattern or Activity

Cytochromes P450 (CYPs) are a superfamily of enzymes containing heme as a cofactor that function as monooxygenase. CYPs are the major enzymes involved in drug metabolism, accounting for about 75% of the total metabolism of xenobiotics. Most xenobiotics are inactivated by CYPs, although some are bioactivated. The detection and quantification of specific metabolite(s) resulting from this metabolization can be a biomarker of exposure. In the case of AR and vitamin K, the pathways of metabolization are not really well-defined. Vitamin K1 metabolism would involve different CYPs. In humans, CYP4F11 and F2 have been identified. The metabolism of warfarin in humans involves CYP2C9, but also CYP1A2, 2C19, and 3A4. In rats CYP1A1, CYP2B1, CYP2C6, CYP2C11, and CYP3A2 (202) are involved in the oxidation of warfarin. The metabolism of other ARs is currently poorly characterized. Nevertheless, the intervention of CYP is probable because of the lipophylic nature of these molecules and the low number of functionalized groups present on the molecules (except for the OH group in position 4, only bromadiolone has a second hydroxy group). In addition, different arguments are present in the literature suggesting a CYP450-dependent metabolism. It has been shown that resistance to difenacoum or chlorophacinone may be caused by increased catabolism through Cytochrome P450 in exposed rodent population (115, 203). Bromadiolone also seems impacted by P450-promoted-catabolism level (117, 204).

Many drugs may increase or decrease the activity of various CYP isozymes either by inducing the biosynthesis of an isozyme (enzyme induction) or by directly inhibiting the activity of the CYP (enzyme inhibition). Many studies deal with Cytochromes P450 as biomarkers of xenobiotic exposure in the liver but also in the blood (easy ante-mortem analysis) thanks to plasma exosomes [e.g., (205–214)]. This enzyme family is really well-studied and known, and methodologies already exist for this kind of application, thus it is a promising approach. However, as Cytochromes P450 responsible of AR catabolism differ according to species and to AR molecules, extensive research is needed to implement this method.

#### Metabolomics and Genomics

Metabolic profiling, also called metabolomics, consists in a screening of a maximum of metabolic parameters, in a fresh biological sample, that should as far as possible be relevant to the precise state or condition of the organism from which the sample come from at the time of the sampling. The usual but non-exhaustive parameters studied are: the evolution of protein expression level, of metabolites, of biological parameters (lipids, proteins, carbohydrates, salts, vitamins…), of cell number and relative types, of redox and acido-basic parameters, etc. The parameters are as well qualitative for example by studying the structure of a molecule or the morphology of a cell, as quantitative, either relatively to a given parameter, either through absolute quantification (215).

One classical use of metabolic profiling is the research of very specific biomarkers for disease or exposure to xenobiotics (216–219). It could therefore be used to discover new specific biomarkers for rodenticides exposure. For example, an ethanolamide plasma lipids regulated by warfarin was found during a metabolomic study and could be used as biomarker of warfarin exposure (220). Moreover, metabolomic is also a screenshot at a given time of many parameters from an organism and combined together, the data can provide a very precise map of the condition of the organism including several parameters (221, 222) converging toward an exposure at anticoagulant rodenticides and permitting differentiation between various levels and nature of exposure as shown already in one study with brodifacoum (223).

The limitation of this method is its current technical difficulty and the requirement of very advanced data analysis (215), including most likely the need of a significant pre-existing comparison database. However, this methodology could become easy, fast, and very reliable through automatization using artificial intelligence combined with deep learning algorithms.

On the other side, genomics is the study of global gene expression patterns. It addresses all genes and their inter relationships in order to identify their combined influence on the organism (224), and relies on DNA or RNA sequencing. A recent study on AR-positive bobcats (*Lynx rufus*) used RNA sequencing on whole blood to investigate genes and cellular processes affected by sublethal exposure to AR. Differential expressions of genes involved in xenobiotic metabolism, endoplasmic reticulum stress response, epithelial integrity and immune function were identified (145). In particular, simultaneous immune dysregulation and disruption of epithelial integrity was suspected to predispose bobcats exposed to AR to opportunistic infections by ectoparasites, e.g., notoedric mange caused by *Notoedres cati*, with an increased mortality rate. Beyond those conclusions, genomics appears as an interesting tool to study all metabolic pathways impaired by AR exposure, allowing for the development of new indicators (e.g., biological parameters related to those metabolic functions) to assess the sublethal effects of AR.

## Conclusion

If rodenticide anticoagulants are currently essentials to manage pest rodent populations, wildlife and domestic animal exposure to AR is a major concern. The exposure level of wildlife is important close to area with human activities. However, it is difficult to assess the consequences of exposure on the non-target animal population, since there are great differences of susceptibilities between species. The biomarkers can be useful tools in this task by highlighting AR effect on non-target species and thus to open the way to an evaluation of pest control practice in order to decrease AR impact. While some biomarkers seem promising, they are currently underdeveloped for AR monitoring purpose. Some of the biomarkers proposed in this study could be developed for systematic use in AR monitoring exposure in the field, while others, which are more complex to implement, could certainly only be developed for research purposes.

## Author Contributions

AR, M-AM, XS, SL, and VL: conceptualization, writing—original draft preparation, and review. All authors contributed to the article and approved the submitted version.

## Conflict of Interest

The authors declare that the research was conducted in the absence of any commercial or financial relationships that could be construed as a potential conflict of interest.
